# Dr. Maclean, on Digitalis

**Published:** 1800-09

**Authors:** 


### Dr. MACLEAN, on DIGITALIS.

[ Continued from pp. 127—141 of our laſt. ]

My ſtrongeſt and moſt vigorous plants grow in a bed or bank ſloping to the ſouth, at the diſtance of ſeveral feet from each other, in an oval, raiſed about a foot and a half above the level of the ſurrounding ground. This was a piece of ground originally detached from the kitchen garden, and raiſed about two feet above its level, (whether by nature or art I know not,) allotted for the growth of ornamental flowers and ſhrubs. The ſoil was naturally light and dry, and it was rendered ſtill more ſo by the addition of a portion of coarſe gravel. But on obſerving the objections urged againſt Horticulture as a general principle, I was deſirous of aſcertaining how far they might be well founded. With this view I directed my gardener, laſt autumn, to tranſplant about 100 plants into the rich ſoil of the kitchen garden, and to add a quantity of gravel ſufficient to looſen its texture. They furniſhed me this ſummer with an abundant crop of leaves, equally ſtrong, healthy, and vigorous, with any I had gathered, although the higheſt ſtems did not exceed four feet. The principal circumſtances I find neceſſary to its ſucceſsful culture in a garden, are, a light dry ſoil, and a ſituation freely expoſed to the ſun and air. The roots of the Fox-glove are extremely ſmall and numerous, and ſeem incapable of penetrating through a denſe ſoil.

The Doctor endeavours to impreſs the belief, however, that he has the authority of eminent botanical writers, from the fifteenth century to the preſent day, in his favour. After repeating his favourite epithets, " low and *damp* ſoil, or a *chalky* ſoil, or a *fat* and *rich* ſoil," which he tells me, " if I conſult the writers upon the natural hiſtory of the plant, will diſcover, are highly detrimental to its growth;" as if I had inſiſted upon theſe being abſolutely neceſſary to it; he obſerves, " that Dr. M. in inſiſting on free expoſure to the air, &c. is in another inſtance deviating widely from the known habitudes of the wild plant;—that botanical writers would inform him, that ſheltered ground is the favourite ſituation of the Digitalis, which courts ſhade, is *always* found in deep hedge-rows," &c. " That I may not be taxed, however, with general or unauthoriſed aſſertion," continues he, " I ſhall conclude theſe remarks by quoting ſome of the firſt, both old and modern writers, on the natural hiſtory of the plant."

" Naſcitur in *montibus*, *umbroſis* et *ſaxoſis* locis. Fuſchius."

" Fox-glove groweth in *barren*, *ſandy* grounds, and under hedges. Gerard."

"It grows only in *gravelly* soils, *rarely* or *never* on those where there are *strata* of *calcarious* earths. Lewis."

"Digitalis. Place; *dry*, *gravelly*, or *sandy* soils;" *particularly on sloping ground.** "Withering."

Now I must take the liberty of telling the Doctor, that none of these authors, nor any that have fallen within my reach, appear to me to have advanced any thing tending to establish what he has asserted.

If Fuschius says, "Nascitur in *umbrosis*," he likewise adds, "*montibus* et *saxosis locis*."

If Gerard tells us, "it groweth under *hedges*," he also mentions, "in *barren*, *sandy* grounds;" Lewes and Withering say nothing of *shade*; and the soils and situations described by them, as friendly to its growth, are in general, the reverse of *shaded*, dry, and gravelly soils; and sloping ground, being, I believe, either elevated or exposed. I can discover nothing like, "*always found in deep hedge-rows*."

If in hedge-rows, on the sides of banks, and *particularly on sloping ground*, the Digitalis be shaded on one side from the sun, it receives his perpendicular rays on the other; if it be sheltered on one hand from certain winds, it will be fully exposed on the other.

It appears evident, therefore, that in regard to shade, even from the authorities he has himself cited, the balance is considerably against him.

Nor does he seem more fortunate in adducing their testimony, as adverse to its culture in a garden. Like all other botanical writers on the natural history of plants, they specify the soils, situations, and habits congenial to them; but does it thence follow that they meant to deny them a place in the garden? If this were the case, many hundreds of plants now in high cultivation in gardens, must necessarily be excluded. The soils mentioned by these respectable authors, may as readily be imitated in the generality of other gardens, as they have been in mine, as far at least as they may be thought necessary. In speaking of the common garden Digitalis, it appears to me, that Dr. D. judges of its virtues entirely from its external appearance, without having put them to the test of experiment; a mode of investigating truth which must often lead to error. There is an obvious difference in the size and heighth of these and the wild plants; yet not so great a variation in their internal qualities. I have had frequent opportunity of making a comparison, not only between the leaves of two parishes

* Why Dr. Drake should have omitted this part of Withering's history, is best known to himself.

rifhes contiguous to that in which the Doctor and his friends loaded their cart, but thofe of feveral gardens, in which no attention was paid to the culture of the plant, more efpecially thofe of the late Mr. Downes, furgeon, of this place, on which my firft trials were made; of Mrs. Young, and S. Brife, Efq. both of Clare; (Dr. D. obliges me to mention names and places.) The firft was not inferior to any I have ever ufed; and an epileptic boy feemed as readily affected by the tincture made from thofe of the latter, as by any other; nor did the medical men among whom it was diftributed, complain of its want of power. The trials, however, were not fufficiently numerous, nor made with that precifion and accuracy neceffary to form a correct judgement of their relative ftrength. One thing, however, I will venture to affirm, that the common garden Digitalis is much more to be relied on than that which is indifcriminately gathered, and in general found in the fhops.

I wifh it to be underftood, that I have entered on this difcuffion concerning the culture of the plant, folely with the view of fhewing the weaknefs and the fallacy of the arguments adduced againft it; for if the authors quoted by Dr. D. and all who have writen on its natural hiftory, had pofitively affirmed that it would not thrive in a garden, I fhould feel myfelf under the painful neceffity of contradicting them.

It gives me pleafure to obferve, that from the attention now paid to its preparation and culture, the medical world are likely to be in poffeffion of the genuine leaves, which alone can enfure fuccefs; for no plant with which I am acquainted, requires more care in the preparation, or attention in the exhibition, than the Digitalis; yet it fhould be added, that none rewards the practitioner more for his pains.

In the analyfis of my remarks, Dr. D. thus proceeds: "The inferences which I have drawn from fome well-known facts in Phyfiology, and to which I am indebted for the ufe of the Digitalis in confumption, appear to me to have been mifunderftood by Dr. M. and to have been objected to on infufficient grounds. In the prefent ftate of medical fcience, no one who has duly cultivated it, and who wifhes to diftinguifh his practice from empiricifm, can or ought to exhibit a new and powerful agent without founding fuch exhibition on the deductions of fcience and analogy." And farther,

" *There are fome modern practitioners, obferves Dr. Darwin, who declaim againft medical theories in general, not confidering that to think is to theorize; and that no one can direct a method of cure without thinking, that is, without theorizing: and happy, therefore, is the patient, whofe phyfician poffeffes the beft theory.*

" Impreffed with the truth of this obfervation, I fhall attempt to prove, and it will be no difficult taſk I think, that what has been termed ' a *theory totally inadmiſſible*,' is but, in ſhort, a ſtatement of facts, drawn from broad experience, and that the only part which merits the appellation of theory, is a mere analogical deduction ariſing from theſe facts. In order to render this more clear, I ſhall ſtate, in numerical order, the different data. 1ſt. Pus is a ſecreted fluid, the conſequence of certain diſeaſed motions of the extremities of the blood veſſels. 2. Hectic fever ariſes only from the matter of an open ulcer. 3. What is termed laudable pus when ſecluded from the air, is neither capable of creating fever, nor, except by its gravity, can it irritate the parts on which it reſts. 4. When pus is excluded from atmoſpheric air, it rapidly attracts oxygen, and an acid of a peculiar kind is generated. 5. Hectic fever is the effect of the abſorption of aerated matter." (Ib. vol. ii. p. 422.) He then proceeds to ſtate the grounds on which theſe data are founded; " and ſhould it be made to appear," ſays he, " that there is juſt grounds for thinking theſe affirmations to be matters of fact, no one will probably deny that the curative proceſſes are legitimately deduced." (Ib. p. 423.)

That I may have unfortunately miſunderſtood the Doctor, " and objected on inſufficient grounds to what he terms, ' well known facts,' is not improbable. Different minds, and ſometimes happily for mankind, form different conceptions of the ſame things and objects. But whether I have done this, " without thinking, that is, without theorizing," is not for me to determine. If I may be allowed this privilege, I own they never ſtruck, nor do they now ſtrike my mind, as laying any claim to the dignified appellation of *facts*, but to be mere phantoms, not of *his*, but chiefly of Dr. Darwin's imagination; which, when we attempt to aſcertain their reality, elude our graſp, and vaniſh like a ſhadow. If this ſhould be proved therefore, it is not likely I ſhould admit, " that the curative proceſſes are legitimately deduced."

As I purpoſe, on a future occaſion, to take a comprehenſive view of this hypotheſis, (which I ſhall ſtill continue to term, " a theory totally inadmiſſible) I ſhall at preſent only offer a few remarks, and contraſt theſe data with what appear to me to approach nearer to facts.

1ſt datum. Pus is a ſecreted fluid, the conſequence of certain *healthy actions** of the extremities of the veſſels which ſecrete it.

2. The

* The learned phyſiologiſt may, at firſt ſight, be ſurpriſed at the term *healthy* being applied to veſſels in a highly morbid or inflamed ſtate. In an Eſſay

2. The matter of an open ulcer is *not* neceſſary to the production of hectic fever.*

3. What is termed laudable pus, either when *ſecluded from* or *exposed to* the air, is neither capable of creating fever, nor, except by its gravity, can it irritate the parts on which it reſts.

4. Although the truth of this datum may be queſtioned, at leaſt when applied to good, thick, bland pus, as found on the ſurface of a healthy wound or ulcer, I ſhall, at preſent, only object to the inference deduced from it; and ſay that,

5. Hectic fever is *never* the conſequence of the abſorption of healthy pus; and it might be added, either in its ſimple or *aerated* ſtate.

The proofs in ſupport of theſe data ſhall be given hereafter, when this ſubject is entered upon more in detail.

Were I to comment upon the above edifying tranſcript from the

---

Eſſay I have had for ſome years, in manuſcript, on Inflammation, nearly ready for the preſs, but which has been ſubjected only to the inſpection of a few medical friends who have intreated its publication, are words to the following effect, which, as applicable to the preſent ſubject, I ſhall tranſcribe: "There are two ſtates or conditions of veſſels in inflammation unfavorable for the ſecretion of good pus; the one *exceſſive*, the other *defective* excitement, which may be termed *morbid* actions. The interference of art will be neceſſary in the one, to abſtract ſtimulus and tone; in the other, to add both, in order to bring the veſſels to the intermediate ſtate, between theſe two extremes, which alone is friendly to the formation of good bland matter. This happy medium I call *healthy*, becauſe it is immediately connected with, and abſolutely neceſſary to, a *healthy* or healing proceſs." A few examples in illuſtration of this are afterwards given, which ſhall, at preſent, be omitted.

This ſtate correſponds with what Mr. Hunter termed *healthy* inflammation.

It really appears as inconſiſtent with the known laws of the animal œconomy, as it is with the rules of ſound logic, to call that action *diſeaſed*, which it is the endeavour of every good ſurgeon to eſtabliſh, from a conviction that without it he will attempt in vain to bring about the healing proceſs.

* Dr. D. ſeems rather unlucky in adducing Dr. Cullen's teſtimony in ſupport of this *datum*, as laid down by him. Even from the paſſage he quotes from his inimitable works, it does not ſeem clear that the words, "*purulency* or *ulceration* of ſome *internal* or *external* part," are neceſſarily and unavoidably to be interpreted into "an open ulcer." But if any doubt ſhould ariſe, it will be completely removed on peruſal of the following paſſage, a few pages farther of the Firſt Lines: "A phthiſis from a ſuppuration in conſequence of pneumonic inflammation, is that which moſt rarely occurs in this climate; and a phthiſis does not always follow ſuch ſuppuration, when the abſceſs ſoon breaks, and diſcharges a laudable pus; but if the abſceſs continue long ſhut, and *till after a conſiderable degree of hectic has been formed*, a phthiſis is then produced, equally dangerous as that from other cauſes. (p. 400).

Nor have I been able to diſcover any thing in Home's excellent Diſſertation on Pus, that bears for his argument, although he quotes it likewiſe in his favour.

Brugman's Diſſertation I have not peruſed.

the Zoonomia, I ſhould be tempted to ſay, there are ſome modern practitioners who declaim againſt experience founded upon experiment and attentive obſervation, and without "thinking, that is, without theorizing, for themſelves, implicitly adopt, without analyſis, every new theory that ingenuity may invent, eſpecially if ſanctioned by the authority of a great name, although it may have no other merit than the charms of novelty to recommend it; and, acting under its influence, they ſeize with avidity upon ſome new remedy, which, from one or two trials, if they chance to be ſucceſsful, they uſher into the world with a character little ſhort of infallibility, and are aſtoniſhed, and not a little piqued, that theſe *unthinking* modern practitioners do not confirm in direct contradiction to common ſenſe, reaſon, and the deliberate reſults of a more enlarged experience:— Unfortunate is the patient who falls into ſuch hands.

No ſucceſsful attempt has yet been made to explain the operation of this plant on the human body: the conjectures hitherto offered, appear in many reſpects vague and contradictory.

If by leſſening the ſuſceptibility of the heart to the ſtimulus of the blood, its contractions be rendered leſs frequent, and the circulation of the blood thus proportionably retarded, it muſt neceſſarily be a direct ſedative to the heart and arteries, Hence, it was employed with this view, and with great ſucceſs, by Dr. Ferriar, in reſtraining inordinate arterial action in active hæmorrhage.

But if, on the other hand, ſerous accumulations be ſpeedily evacuated by its means in dropſy, it will, by parity of reaſoning, be contended, that it acts by a direct ſtimulant operation on the lymphatics and emulgent veſſels; conſequently, that it poſſeſſes directly ſedative and ſtimulant effects on the body at the ſame time. In this manner has its ſalutary agency in the cure of conſumption been explained; and this idea has been carried ſo far, that it is imagined, the action of the abſorbents is ſo powerfully excited, as not only to take up the pus as ſoon as ſecreted, but even the tubercles themſelves.

Reaſoning thus, however, muſt ever lead to endleſs difficulty and embarraſſment. It is, I preſume, as contradictory to the principles of ſound phyloſophizing, as to the known laws of the animal machine, to aſſume it as a fact, that any ſubſtance is capable of producing two directly oppoſite effects at the ſame time on different parts of the ſame ſyſtem of veſſels, Whatever may be its mode of action, it muſt neceſſarily be the ſame, under ſimilar circumſtances, on all parts; the heart, vaſcular, and abſorbent ſyſtem. It is, by arguing in this manner, that there has been ſuch a contrariety of opinion on the *modus operandi* of opium; ſome regarding it as a ſedative, others as as a ſtimulant, according as they took a more or leſs comprehenſive view of its effects on the body.

On attentively obſerving the action of the Digitalis, it ſtruck me as a ſingular circumſtance, that in all the caſes in which it was preſcribed, (now pretty numerous) unaccompanied with ſigns of ſerous effuſion, I could diſcover no ſenſible increaſe of the urinary ſecretion, nor any of the effects conſequent on the long continued uſe of large doſes of other diuretic medicines, even when the habit had been for ſome weeks under its full influence, although the colour ſeemed at times materially changed: Hence I inferred, that its operation as a diuretic, was immediately and neceſſarily connected with watery accumulation. But, on reflecting that the generality of diuretics increaſe the flow of urine, more or leſs, in health as in diſeaſe, moſt probably by a direct ſtimulant operation on the kidneys, I was unavoidably led to conclude, that the Digitalis poſſeſſed no ſuch effect; and that, as a ſucceſsful remedy in dropſy, its action was chiefly, if not ſolely, confined to the abſorbents. This opinion is ſtrengthened, if not confirmed, by the complete ſucceſs which followed its uſe in two caſes of hæmaturia, attended with dyſuria, and extremely painful micturition, in which I preſcribed it. And, if well founded, of which I have little doubt, it overturns Dr. Ferriar's notion, that "we are debarred from its uſe in one caſe of local increaſed action, that of diabetes." This ingenious author acknowledges his inability to explain theſe *apparently* oppoſite effects, and leaves the queſtion, *ſub judice*; for, ſays he, "I feel it impoſſible to explain this phenomenon at preſent." (p. 13.) And again: "This double effect of Fox-glove, however perplexing in theory, is extremely beneficial in practice; when it takes place, it adds, in hæmorrhages, a mode of natural evacuation, ſufficiently efficacious to relieve plethora, without directly debilitating the ſyſtem." (p. 15.)

But if the view in which I have conſidered this part of the ſubject ſhould be correct, the difficulty is completely removed. It cannot be denied, that by its uſe the lymphatics are placed in a condition of taking up ſpeedily from cavities, inordinate quantities of effuſed fluid; yet, I truſt, I ſhall advance ſome ſteps in proving, that this is not by a ſtimulant operation; indeed, if its ſedative effects on the heart and arteries be admitted, this idea muſt neceſſarily be rejected. The manner in which the functions of the abſorbents are impaired in dropſy, and ſome other diſeaſes, ſeems as yet very imperfectly known; whether by paralyſis, inverted or retrograde motion, or the like, has not been ſhewn; but in whatever way this may be, the the Digitalis poffeffes, in an eminent degree, the power of reftoring them.

I fhall be happy in finding my fentiments, in this refpect, coincide with thofe of Dr. Kinglake. In one of his ingenious Communications he obferves, " My opinion of the *modus operandi* of Digitalis is founded on a principle of lymphatic circulation, that wholly rejects the prevailing but gratuitous notion of the lymphatic veffels, abforbing and propelling by virtue of any independently active or contractile power, &c." (*Ib. vol. iii. p.* 125.) Yet, I fee difficulty in reconciling a direct fedative operation, which I fuppofe this herb univerfally to poffefs on the body, with the fundamental principles of the Brunonian doctrine, which he has with fo much ability fupported. Permit me in this place to obferve, in reply to his very candid and liberal remarks on my former paper, that naufea has, with me, never interfered with the falutary agency of Digitalis in confumption. In nearly all the cafes in which it fucceeded, (now a confiderable number) the ftomach has been more or lefs affected; but when this organ, and the general habit were readily difordered, its fuccefs was very uncertain.

I wifh, in a particular manner, to draw the attention of the medical world to this univerfally fuppofed action of the Foxglove on the kidneys; as on the point of view in which I have been led to confider the fubject, are founded the moft important practical inferences, efpecially in dropfy, which fhall hereafter be confidered in detail. With few exceptions, I obferve the reports of others, who have ufed it in cafes unattended with watery accumulations, accord with my own obfervations. Some fay nothing of its diuretic effects, while others pofitively affirm, that no fuch were obferved. But, as many cafes of confumption are conjoined with anafarcous fwellings, or water in the cavities of the cheft, the practitioner may, in this refpect, be fometimes deceived. " I fhall have occafion," fays Dr. Ferriar, " to mention fome cafes, in which thefe two operations were combined in a remarkable degree." But I have in vain looked for thefe cafes in his laft Effay.

I am happy in having it in my power to adduce an obfervation of Dr. Drake, in fupport of the opinion I have been advancing. In endeavouring to maintain the fuperiority of the tincture over the powder in confumption, he fays, " a material difference too, may be obferved in their mode of operation; for whilft the powder ufually increafes, and changes in point of colour, the evacuation of urine, I have *never*, but in one inftance, and that but in a flight degree, perceived a fimilar effect from the ufe of the tincture." If he had *never* ufed the powder in confumption, how could he obferve any difference difference in their mode of operation? And if he judged only of its effects in dropfy, the difference he mentions was a natural and a neceffary confequence.

I venture now, from ample experience, and without the fear of contradiction, to affirm, that no fuch difference will be perceived by an unbiaffed mind, in their mode of operation, in confumption.

I fhall take my leave of the Doctor and the fubject of the Digitalis, for the prefent, by affuring him, that, as the inveftigation of truth, the advancement of medical fcience, the leffening the fum of human mifery, are, and have been, my fole objects in the prefent and in former difcuffions; however averfe I may be to controverfy of every kind, and unequal as I am to the tafk, when my humble talents are oppofed to thofe of the author of "Literary Hours," I fhall not fhrink from any farther enquiry that may tend to promote thefe important ends.

*July* 1, 1800.

P. S. Since writing the above, while attending with Mr. Travis, furgeon, of Eaft Bergholt, at Higham, one of the parifhes which fupplied me liberally with Digitalis laft year, I was defirous of feeing the plant with this gentleman, more efpecially as he affifted, about a week before, as well as laft year, in collecting the leaves with Dr. Drake. We were conducted to an eminence about two miles from the houfe where we were attending, and as many from Eaft Bergholt. On the fides of feveral fandy barren hills, and in adjoining hedge-rows, we perceived fome hundreds of plants, a few of which feemed taller than any I had ever feen; but fo fcantily fupplied with thin, narrow, meager leaves, that a boy who accompanied us, was fome hours in filling a fmall bafket. Mr. Travis informed me, however, they were inferior in every refpect to thofe of his gathering, particularly fome he and the Doctor found in a *fhaded* lane. When dried, they were fhrivelled and difcoloured, and apparently far inferior to thofe of the two gardens already mentioned, as well as that of R. Andrews, Efq. Receiver General for the county of Effex, on which fome experiments fhall foon be made. Were I to truft to thefe, I am perfuaded I fhould be difappointed. In hot, dry fummers, like the prefent, when every vegetable growing on fandy foils and elevated fituations is foon dried up, it is probable, the Digitalis may be found in higher perfection in *fhaded* lanes, where it has more moifture, and is defended from the fcorching rays of the fun.

I have lately fent two quarts of the tincture, and a few ounces of the dried leaves of my own growth, to Apothecaries Hall,

Hall, as ſpecimens to be referred to, and uſed by ſuch of the Faculty in town as may be intereſted in the ſucceſs of this plant; and may wiſh to ſee and make experiments upon the genuine preparation.

The mixture is made in the proportion of one ounce to eight; and I beg again to obſerve, that this quantity furniſhes more of the active parts of the leaf, than the ſpirit is capable of taking up and retaining; after ſome weeks digeſtion, a conſiderable portion of extract remaining behind on the filtering paper. The ſubject is now, however, in much better hands. In a letter, the learned Preſident of the College has done me the honour of writing me lately, after politely ſaying, "that I deſerve the thanks of the Faculty, for the attention I have paid to a drug of great efficacy;" adds, "that he will loſe no opportunity of mentioning my improvements to the gentlemen of the college, and of trying the effects of my tincture."

