# Errata

**Published:** 1800-10

**Authors:** 


240, l. 12, for *excluded from*, read *exposed to*.

—— l. 2, Note, dele *morbid or*.

246, l. 5, for *mixture*, read tincture.

